# Development of a multipotent diagnostic tool for chest X-rays by multi-object detection method

**DOI:** 10.1038/s41598-022-21841-w

**Published:** 2022-11-09

**Authors:** Minji Kang, Tai Joon An, Deokjae Han, Wan Seo, Kangwon Cho, Shinbum Kim, Jun-Pyo Myong, Sung Won Han

**Affiliations:** 1 School of Industrial and Management Engineering, Korea University, Anam-ro 145, Seongbuk-gu, Seoul, 02841 Korea; 2 Division of Pulmonary and Critical Care Medicine, Department of Internal Medicine, Yeouido St. Mary’s Hospital, College of Medicine, The Catholic University of Korea, Seoul, Korea; 3 Doctors on the Cloud, Seoul, Korea; 4 Division of Pulmonary, Allergy, and Critical Care Medicine, Department of Internal Medicine, Changwon Fatima Hospital, Changwon, Korea; 5 Division of Pulmonary, Allergy, and Critical Care Medicine, Department of Internal Medicine, Andong Sungso Hospital, Andong, Korea; 6 Department of Occupational and Environmental Medicine, Seoul St. Mary’s Hospital, College of Medicine, The Catholic University of Korea, Banpodae-ro 222, Seocho-gu, Seoul, 06591 Korea

**Keywords:** Image processing, Machine learning, Medical research

## Abstract

The computer-aided diagnosis (CAD) for chest X-rays was developed more than 50 years ago. However, there are still unmet needs for its versatile use in our medical fields. We planned this study to develop a multipotent CAD model suitable for general use including in primary care areas. We planned this study to solve the problem by using computed tomography (CT) scan with its one-to-one matched chest X-ray dataset. The data was extracted and preprocessed by pulmonology experts by using the bounding boxes to locate lesions of interest. For detecting multiple lesions, multi-object detection by faster R-CNN and by RetinaNet was adopted and compared. A total of twelve diagnostic labels were defined as the followings: pleural effusion, atelectasis, pulmonary nodule, cardiomegaly, consolidation, emphysema, pneumothorax, chemo-port, bronchial wall thickening, reticular opacity, pleural thickening, and bronchiectasis. The Faster R-CNN model showed higher overall sensitivity than RetinaNet, nevertheless the values of specificity were opposite. Some values such as cardiomegaly and chemo-port showed excellent sensitivity (100.0%, both). Others showed that the unique results such as bronchial wall thickening, reticular opacity, and pleural thickening can be described in the chest area. As far as we know, this is the first study to develop an object detection model for chest X-rays based on chest area defined by CT scans in one-to-one matched manner, preprocessed and conducted by a group of experts in pulmonology. Our model can be a potential tool for detecting the whole chest area with multiple diagnoses from a simple X-ray that is routinely taken in most clinics and hospitals on daily basis.

## Introduction

Chest X-ray (CXR) is a general practice for evaluating the chest anatomy, which is routinely interpreted by chest radiologists^1^. Tremendous amount of CXR have been and will be taken all over the world and it will remain to be the most frequently performed radiologic images for another couple of decades^2^. Nevertheless, there are many obstacles for getting proper interpretation of CXR. Errors in interpreting CXR images are relatively common in general practice due to the large amount of daily interpreting works given to radiologists, leading to negligence errors^3^. It is also due to lack of specialists and experts as well as their low performance which is explained by the Pareto’s Law: a various range of performance of experts and extremely high proportion of low performers^4^. This kind of errors was reported in about 30% of all CXR reports, which is approximately 40 million cases per year^3,5^. Furthermore, radiologists have only one tool, human naked eyes, that show very limited ability for distinguishing spatial resolution of high quality CXR images^6,7^.

The rise of computer-aided diagnosis (CAD) for medical imaging techniques such as classification, detection, and segmentation began for overcoming the above-mentioned weakness of CXR^8–10^. A lot of CAD models for CXR were developed over the past 50 years^11–14^. Even though the techniques of CAD have advanced far beyond the physiologic cognitive functions of human (for instance, the deep learning algorithm), there are still many limitations to the CAD models in terms of when performance, reproducibility, accuracy, and versatility, when it comes to applying them to the real clinical fields^13,15,16^. One of the most important reasons for the weakness is poorly labeled data when training the CAD models^17^. Most of the CAD models developed in previous were trained by labeled data which contained a single or a few image descriptions with poor quality and accuracy^2^. The CAD models developed by such data inevitably show poor diagnostic performance. In order to overcome this innate weakness, many later studies solved their problems by inputting a tremendous amount of data for training^18^. However, there is a saying in developing deep learning fields that “Garbage in, Garbage out”. Subsequently, the importance of data labeling by professional medical experts has grown. Nowadays most researchers deal with studies on the CAD for computed tomography (CT) more than that for the CXR^19,20^. However, CT scans are neither the first test nor an image tool routinely used in practice when evaluating pulmonology and cardiology areas. That is because the CT scans are expensive (about 10 times more than CXR in South Korea), with higher radiation exposure (10 millisievert for chest CT scan is 100 times higher than that of CXR), and less accessible (difference between urban vs. rural sides and developed vs. developing countries). Because of these reasons, most medical doctors, whether they are novice or experts, prescribe CXR first, even after several decades have passed since the first development of CT. The CXR is simple, cheap, and one of the easiest ways to understand the chest area.

In order to overcome the weakness of CXR and reflecting the strong points of CT scan, we planned to develop a multipotent diagnostic CAD model by applying multi-object detection techniques by using preprocessed data which was matched with chest CT description one-on-one to each designated region of CXR image by a group of experts in pulmonology. And, by doing so, we planned to extend the range of diagnoses in a single model and widen the possibility of real-world use of the model by clinicians.

## Materials and methods

### Data source and preprocessing

**Figure 1:**
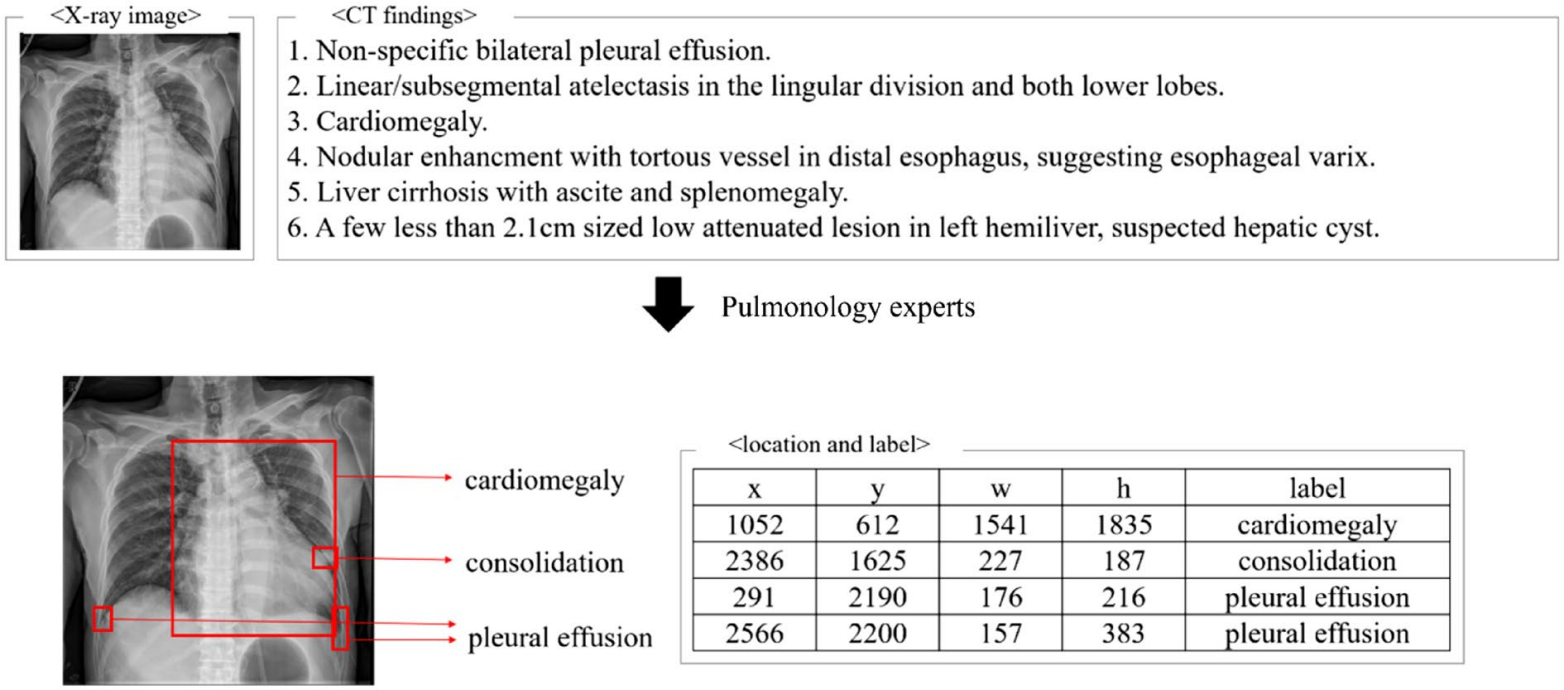
The schematic flow of data preprocessing. Each lesion of X-ray is labeled with a bounding box on the chest image by pairing one-to-one with the chest computed tomography description which was taken on the nearest time at exact same day. Each bounding box consists of the upper left x, y coordinates, width, height, and lesion class values. Bounding box was measured by trained pulmonology experts.

For developing universal chest X-ray reading program, we required techniques that can detect all classes of lesions which was studied in previous articles^2^. Moreover, it is required that the lesion which was only seen in CT scan must be included in this program. For that, the X-rays and CTs which were performed on same days were extracted, and these were taken at Yeouido St. Mary’s Hospital, the Catholic University of Korea between 2017 and 2018. If multiple events of X-ray were found, the one with the closest time to CT was selected. X-rays that have matching CT scans but with no final reports of CT findings had to be excluded. If X-rays were taken right after procedures, such as insertion of chemo-port, percutaneous chest drainage, or Levin tube, and did not correlate with CT description, they were excluded from our final data collection. Then, each chest X-ray lesion was matched one-on-one to the associated CT finding with bounding box locating the lesion. The bounding box showed the information of suspected lesions into x-coordinate (upper left x), y-coordinate (upper left y), transverse length (width), vertical length (height), and label^21^. All these data preprocessing steps were proceeded and co-validated by trained five pulmonology experts (Fig. 1).

### Structures of multiple object detection models

Traditionally, object detection was a method for locating objects within an image by defining the coordinates of a bounding box and by predicting the appropriate category for the detected object^22^. The three key ideas of our model were as follows. First, we used ResNet101 as the backbone to extract features from the images^23^. Then, we located the position of the features within the feature maps and predicted their coordinates and types of class. We tested them by using both the Faster R-CNN and the RetinaNet^24,25^. Finally, the bouncing box was selected by using the non-maximum suppression (NMS) algorithm.

### ResNet101

**Figure 2:**
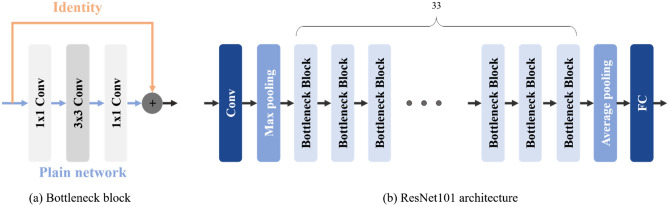
The layer structure of bottleneck block and ResNet101. (**a**) Bottleneck block has a skip connection by adding identity to plain network. (**b**) ResNet101 consists of 33 bottleneck blocks.

ResNet101 was 2015 ILSVRC-winning network developed by Microsoft^26^. It introduced a residual framework to facilitate learning in deeper neural networks with more layers than conventional deep neural networks. The residual framework used skip connections for CNN. It was an effective way to train residuals minus the identity in a plain network. We used a bottleneck structure with three layers between skip connections for low time complexity and model size compared to projection. The network also sought to improve the gradient vanishing problem by using ReLU as an activation function and batch normalization. As shown in Fig. 2, we used ResNet101 as the backbone for the Faster R-CNN and applied transfer-learning by taking pre-trained weights for the Pascal VOC dataset^27^.

### Faster R-CNN and RetinaNet

**Figure 3:**
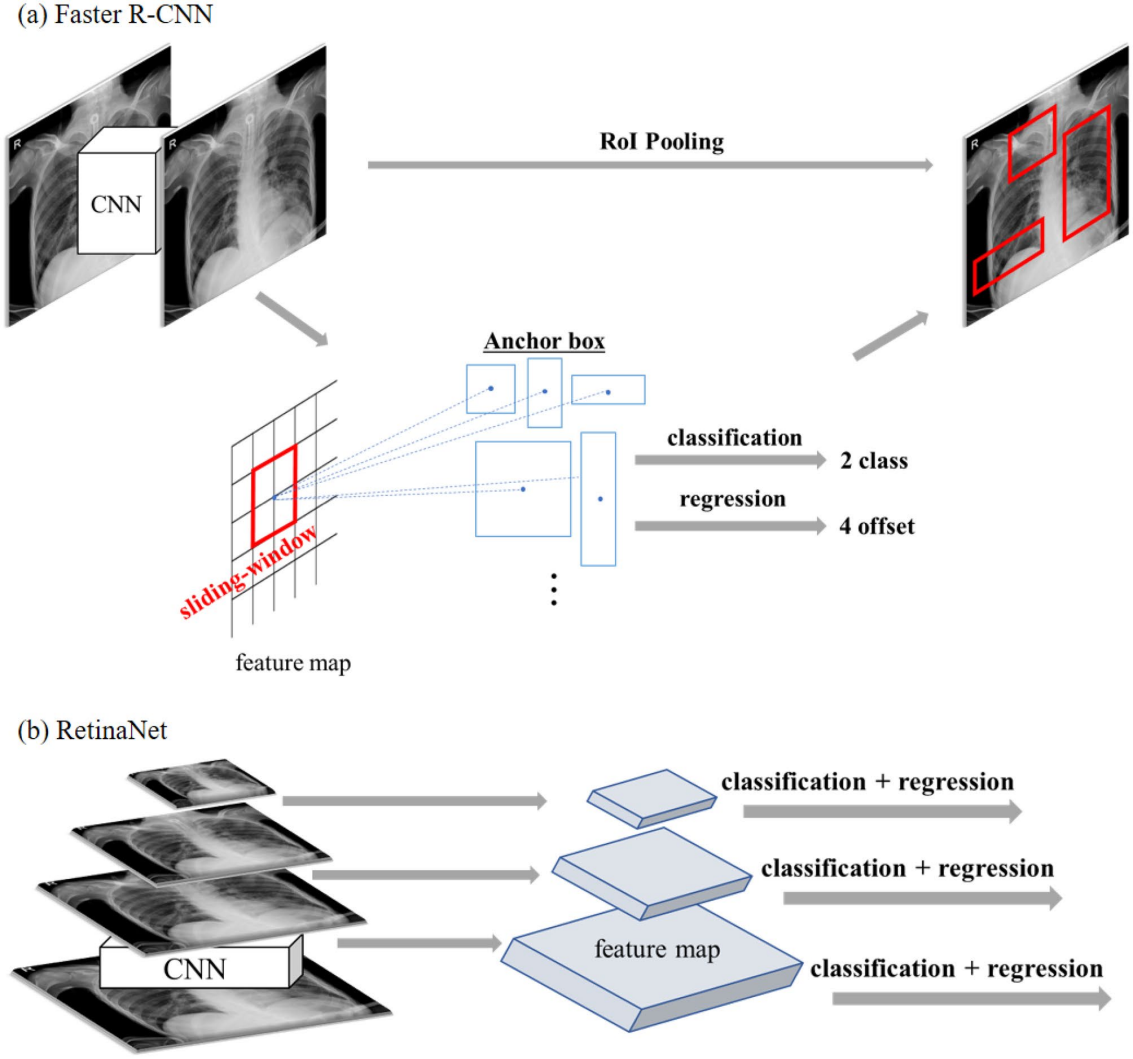
Region proposal network in Faster R-CNN architecture and RetinaNet. Illustrations of two-stage method (faster R-CNN) and one-stage method (RetinaNet) were described in Figure 3. Each method is defined by whether or not the models are divided by the classification and regression steps. (**a**) Faster R-CNN is performed by multiple region proposals by a sliding-window, so-called anchor box, on the extracted feature map. Each anchor box performs classification for object detection and regression for adjusting the position of the box. Then, each box predicts object classes with the bounding box. (**b**) RetinaNet performs the classification and finding coordinates simultaneously. It changes the loss function of captured candidates into background class, and the weight of real objects, which was difficult to classify before, is increasing.

The Faster R-CNN is the representative model of two-stage method in the object detection, which are divided by distinguishing class of images (classification) and finding location (region proposal)^24^. It predicts multiple region proposals by a sliding-window, so-called anchor box, which varies in aspect ratio and size. Each anchor box performs classification for determining whether objects exist or not, and regression for adjusting the position of the box. After these steps, each box is categorized into the predicted object classes with the coordinates of the bounding box. The RetinaNet is one of the main models of the one-stage method, which performs the classification and finding coordinates simultaneously^25^. One-stage model trains candidates up to thousands and then captures the large portion of candidates as the background class which is easy to classify. By changing the loss function, the weight of real objects increases, which was difficult to classify before. It allows the training model to focus on real objects. The details were described in Fig. 3.

### Non-maximum suppression (NMS)

There were many overlapped bounding boxes predicted by the above-mentioned models. It was considered as the same box if the degree of overlapping was sufficient for making decision. NMS was selected by the method for choosing the correct bounding box from other predicted ones. First, the algorithm sorted the bounding boxes in the order of confidence for each class. Then, the intersection of union (IoU) was calculated by comparing the highest scored bounding box with the other boxes. If the IoU was higher than a certain threshold, bounding box with the highest score was selected. The other boxes that overlap with the box with the highest score were removed^28^.

### Study setting

In this study, approximately 80% (236 people) of the dataset were used as learning data and the rest of 20% (54 people) were allocated to the test data. We used the stochastic gradient descent considering momentum as an optimization method. The learning rate was set to decrease with a weight decay of 0.0001 with a momentum of 0.9 as learning progresses, starting with a learning rate of 0.001. We performed training with 5000 epochs, and the epoch that showed the highest accuracy was used to produce the results. Each batch was trained on a region-of-interest basis and the batch size was 128. The scales of anchors used in Faster R-CNN were 8, 16, and 32, and the ratios are 0.5, 1, and 2. The inference time of Faster R-CNN was 0.198 s which was calculated as 5 frames per second (FPS). That of RetinaNet was 0.145 s which was calculated as 7 FPS. Intel i7-7700 CPU, 64 GB RAM, and two NVIDIA GeForce GTX 1080Ti GPUs were used in model training.

### Statistics

True and false positive were defined by the value of IoU. If the IoU of the predicted and actual regions exceeds a certain threshold, the classifier's performance is determined to be correct, and it is defined as true positive. Conversely, if the IoU value is less than the threshold, the result is defined as false positive. We calculated sensitivity, specificity, accuracy, and average precision (AP) with a confusion matrix between the predicted and the actual regions, which were compared by chi-square analyses. The *p* values less than 0.05 were considered statistically significant. The cumulative value was determined by listing the detected regions in the order of confidence score. As the regions were listed, we figured a precision-recall curve with the accumulated values and computed the AP from the area bellow.

$$Sensitivity= \frac{True\, Positive}{False\, Negative+True\, Positive}$$

$$Specificity= \frac{True \,Negative}{True\, Negative+False \, Positive}$$

$$Accuracy= \frac{True \, Positive+True \, Negative}{True \, Positive+True \, Negative+False \, Positive+False \, Negative}$$

$$Average \,Precision= \frac{1}{11}\sum_{Recall}Precision(Recall)$$

### Ethical approval and consent to participate

Current study was conducted according to the Helsinki declaration. It was approved by the Ethics Committee of the Catholic University of Korea Yeouido St. Mary’s Hospital (SC19RIS0166). Informed consents were waived due to retrospective setting of the study.

## Results

### Data description

A total of 12 CT-based diagnostic labels were defined during the data preprocessing: pleural effusion, atelectasis, pulmonary nodule, cardiomegaly, consolidation, emphysema, pneumothorax, chemo-port, bronchial wall thickening, reticular opacity, pleural thickening, and bronchiectasis. Overall, 1439 bounding boxes were extracted from 290 training dataset. Among them, 1148 bounding boxes were used in training model and rest of 291 bounding boxes were used in the test set. The details of the used data were summarized on Table 1.

**Table 1:** Summary of one-to-one CT matched chest X-ray bounding box data.

Chest X-ray findings	Total	Training set	Test set
Pleural effusion	173	140	33
Atelectasis	174	133	41
Pulmonary nodule	241	192	49
Cardiomegaly	31	22	9
Consolidation	230	190	40
Emphysema	85	69	16
Pneumothorax	34	27	7
Chemo-port	27	19	8
Bronchial wall thickening	147	127	20
Reticular opacities	207	157	50
Pleural thickening	23	20	3
Bronchiectasis	67	52	15
Total	1439	1148	291

The expert-labeled data was described. In the training set, approximately 80% of data were included. Its range is various from 20 to 192 bounding boxes.

### Results from each model

**Figure 4:**
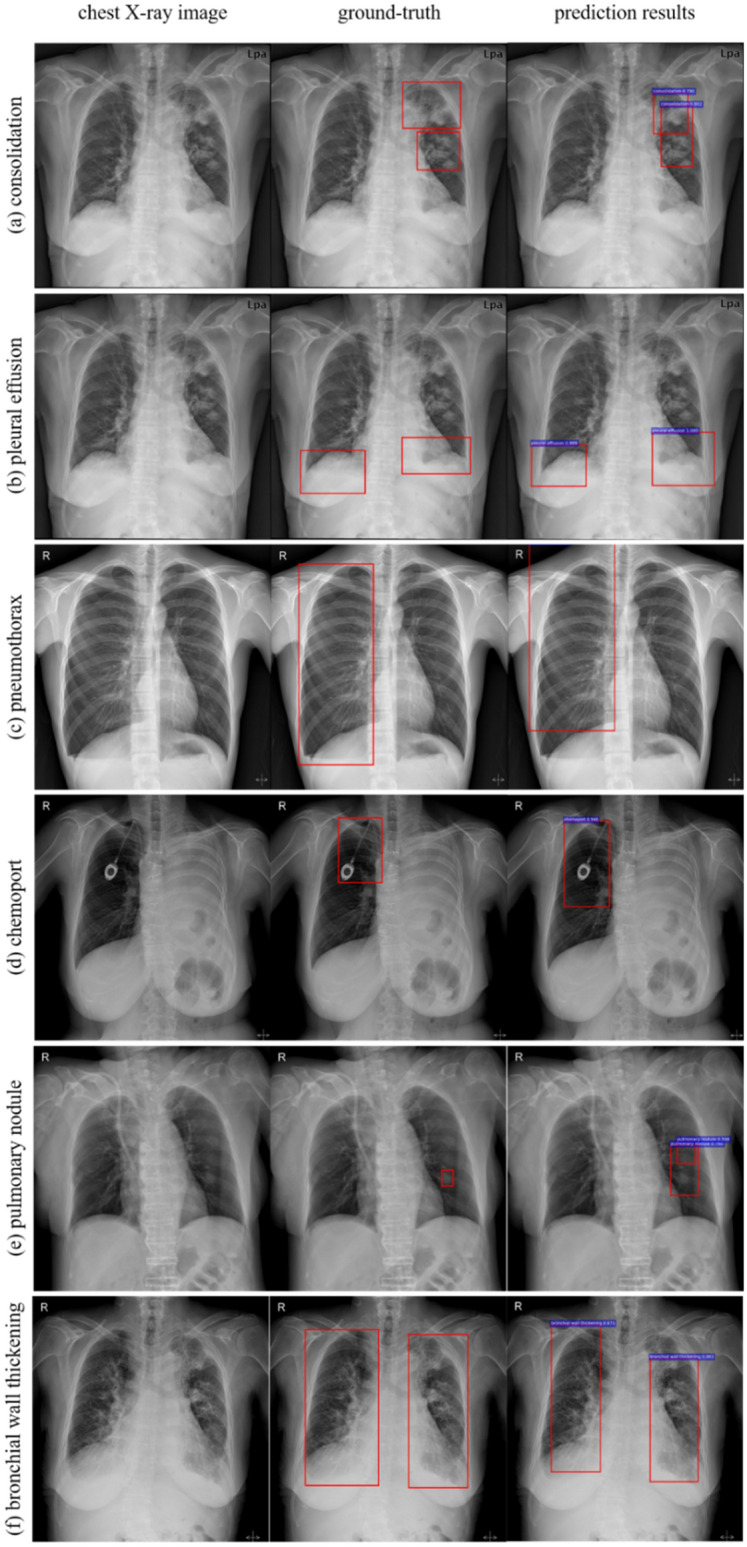
Examples of prediction results of multi-object detection in this study. Examples of the predicted results are shown in Figure 4. Overall, twelve classes are described in this model, such as pleural effusion, atelectasis, pulmonary nodule, cardiomegaly, consolidation, emphysema, pneumothorax, chemo-port, bronchial wall thickening, reticular opacity, pleural thickening, and bronchiectasis. From left to right, actual chest X-ray image, ground-truth (expert-described data labels), and results from prediction model are shown.

Examples of prediction results were showed in Fig. 4. We compared the outcomes of learning by Faster R-CNN based model and by RetinaNet based model. They showed the different tendency of results. Overall sensitivity is higher in faster R-CNN based model (53.3%) than that of RetinaNet based model (13.1%). The specificity is higher in RetinaNet based model (94.3%) than that of faster R-CNN based model (24.5%). In the subgroup of faster R-CNN based model, the class of cardiomegaly and chemo-port showed excellent sensitivity (100.0%, both), fair specificity (74.5% and 74.6%), and fair accuracy (75.6%, both). The classes like bronchial wall thickening, pleural effusion, pleural thickening, and emphysema also showed fair results in sensitivity. On the other hand, the classes like atelectasis, pulmonary nodule, and pneumothorax showed lower performance in terms of sensitivity, specificity, and accuracy. Some values, which were usually described in chest CT, such as bronchial wall thickening, reticular opacity, and pleural thickening showed relatively moderate to fair results in sensitivity. In the subgroup of RetinaNet based model, all classes showed high specificities but with lower sensitivities. When we compared the two models, faster R-CNN model showed better sensitivity in detection of suspected lesion and RetinaNet based model was better for distinguishing the normal values (*p* < 0.01) (Table 2). Overall examples of images of each class were summarized on Supplementary Figure S1.

**Table 2:** Performance summary of Faster R-CNN and RetinaNet.

Algorithm	Faster R-CNN				RetinaNet			
Classification	Sensitivity (%)	Specificity (%)	Accuracy (%)	mAP	Sensitivity (%)	Specificity (%)	Accuracy (%)	mAP
Pleural effusion*	72.7	35.8	38.8	41.6	24.2	91.3	79.9	15.4
Atelectasis*	29.3	17.1	17.7	10.0	4.9	96.8	77.9	2.1
Pulmonary nodule*	32.7	9.1	9.8	5.3	2.0	98.7	75.6	0.0
Cardiomegaly*	100.0	74.5	75.6	31.0	22.2	97.5	93.4	0.0
Consolidation*	50.0	17.7	19.3	16.1	2.5	96.3	77.5	1.4
Emphysema*	68.8	37.6	38.8	5.1	25.0	92.6	86.6	9.2
Pneumothorax*	42.9	55.5	55.2	29.5	0.0	96.3	92.3	0.0
Chemo-port*	100.0	74.6	75.6	93.9	37.5	99.3	96.3	37.5
Bronchial wall thickening*	75.0	26.2	27.9	20.1	50.0	75.5	73.1	20.8
Reticular opacities*	54.0	14.8	16.9	5.7	14.0	94.3	74.9	9.2
Pleural thickening*	66.7	46.5	46.7	0.6	0.0	100.0	98.1	0.0
Bronchiectasis*	53.3	21.7	22.4	2.1	0.0	97.5	89.1	0.0
Total*	53.3	24.5	25.7	21.8	13.1	94.3	83.7	8.0

Performance results were summarized on this table. Faster R-CNN showed higher sensitivity than RetinaNet. Some classes such as cardiomegaly or chemo-port showed 100% sensitivity. Others, which were not easily seen in chest X-ray compared to those of computed tomography, such as pleural effusion, emphysema, pleural thickening, and bronchial wall thickening were described by the faster R-CNN model. Compared to those of faster R-CNN, RetinaNet model showed higher specificity and accuracy. However, this model showed poor results on sensitivity, which were not good outcome for abnormal object detection. *p < 0.01 (results of faster R-CNN were compared to those of RetinaNet).

## Discussion

A tremendous amount of chest X-rays are generated day-by-day. However, the number of chest radiologists is limited. And that is why the outsourcing industry of radiologic area is emerging today. The need of CAD program for chest area is also rising. However, the value of CAD for chest X-ray have been undervalued because of its limitation, such as need of a large baseline training data due to its lower imaging quality compared to the CT, both in image resolution and data labeling^2^. The recent development of CAD for chest CT has improved the imaging quality problem. However, CAD for chest CT has weakness in several areas. First, it is still not the first radiologic tool for evaluating chest area and is not evenly distributed around the world, especially in developing countries. Second, it has radiation exposure problem compared to chest X-ray, as much as 70 times higher exposure. Third, it is still unipotent for a few numbers of diseases only. Fourth, the excessive amount of data from CT makes it rather difficult when it comes to feeding the large data to deep learning algorithm. Also, there are very few reliable datasets for the training.

This study showed the interesting results regarding the above-mentioned aspects. First, we showed the versatility in use by using multi-objection detection model which was developed for multipotent ability to diagnosis (Fig. 4). For the versatility in use, in real-world medicine, the assistant program must have the ability to describe the chest X-ray in more detail than just a single word or phrase. Some classes, such as cardiomegaly or chemo-port, showed 100% sensitivity given the small amount of training set. Other classes which were usually described in chest CTs but not in chest X-rays, such as bronchial wall thickening, reticular opacity, and pleural thickening, raised the promising possibility to use this multipotent program for describing the chest area. Even though the labeled data in the training set was relatively small than that of other previous studies, it showed fair results which may be applied to various areas. Second, we also showed that the application of two-way method gave more benefits than that of one-way method for detecting suspected pathologic lesions by the multi-object detection. Faster R-CNN (two-way method) showed higher values in detecting the suspected lesion (sensitivity) than RetinaNet (one-way method). Nevertheless, the RetinaNet showed the superiority in detecting the normal lesion compared to that of faster R-CNN (specificity).

There were several limitations to this study. First, the overall performance of models was not high enough. We trained the model with a small amount of data, and this could be the most explainable reason to the low performance. However, considering that it was conducted as a pilot study, it showed the potentials of multi-object detection by using the novel approach with one-to-one matching of CT findings to CXR. In our future study, we planned to gather more well-labeled preprocessed data in order to overcome this small data issue. Second, there were limitations about the bounding boxes. The bounding boxes could only represent the geographic information with square-shaped boxes. As results, there were problems with sensitivity because the square bounding boxes inevitably contain non-pathologic normal part of chest images. The future study will need to improve the outcomes by using different shapes of the bounding boxes.

Conclusively, we showed the possibility of developing the CAD models for describing the whole chest area by multi-object detection methods. It can become valuable has the value as a part to the automated reading program, especially in primary care. Moreover, we used the novel approach by using the CT findings/description in one-to-one matched manner to the CXR image processed by the experts in pulmonology. Best to our knowledge, this was the first study revaluing the simple chest X-rays used for developing a novel CAD model as an all-rounder diagnostic tool.

## Supplementary Information

Supplementary Information.

## Data Availability

Researchers may send reasonable requests for access to the datasets used in this study to the corresponding author.
