# Supplementary material for: Development of a multipotent diagnostic tool for chest X-rays by multi-object detection method
Source: Sci Rep. 2022 Nov 9;12:19130. doi: 10.1038/s41598-022-21841-w (PMC9646869; doi:10.1038/s41598-022-21841-w)

# Figures

Supplementary Figure S1. Examples of images of each class

S1-1) Pleural effusion


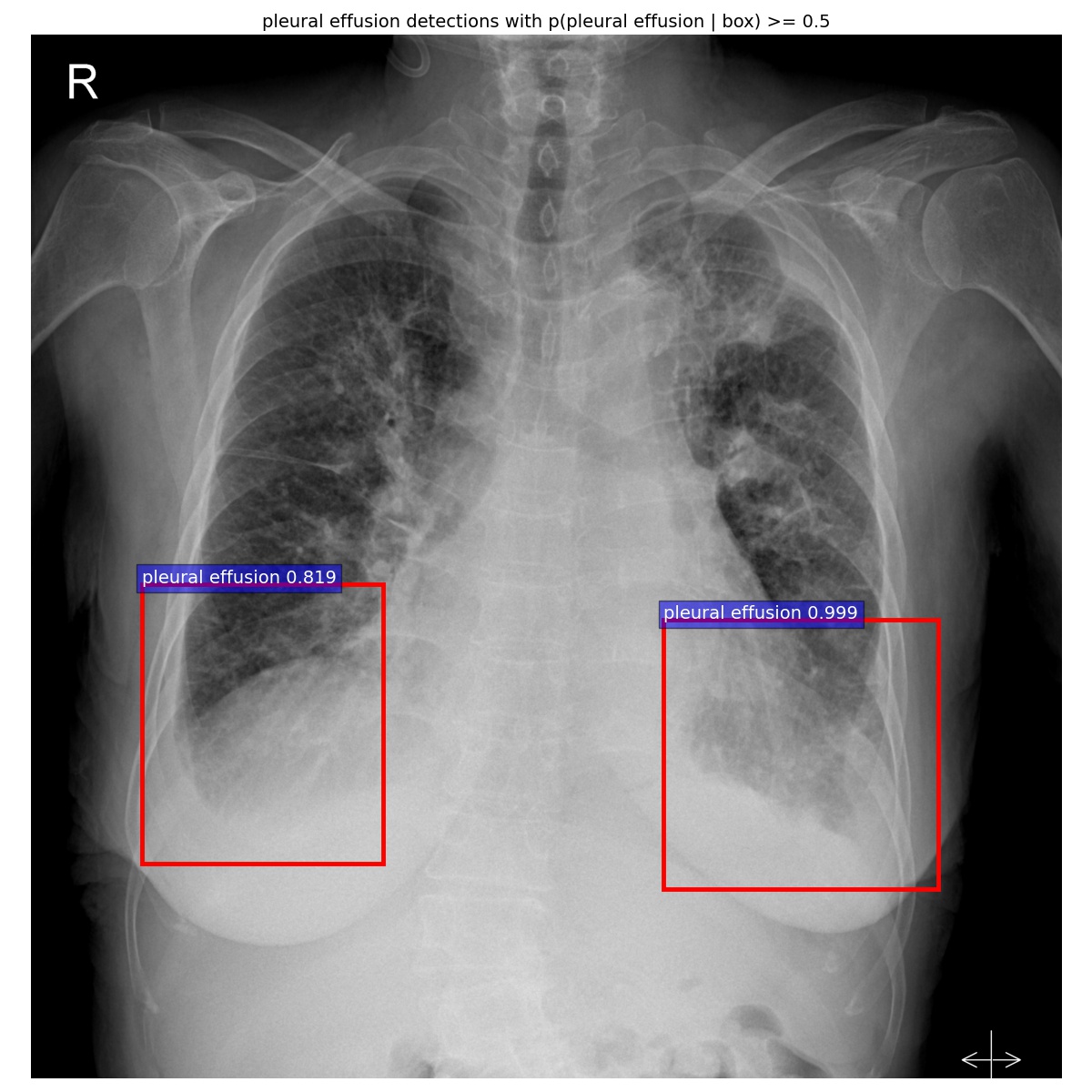


S1-2) Atelectasis


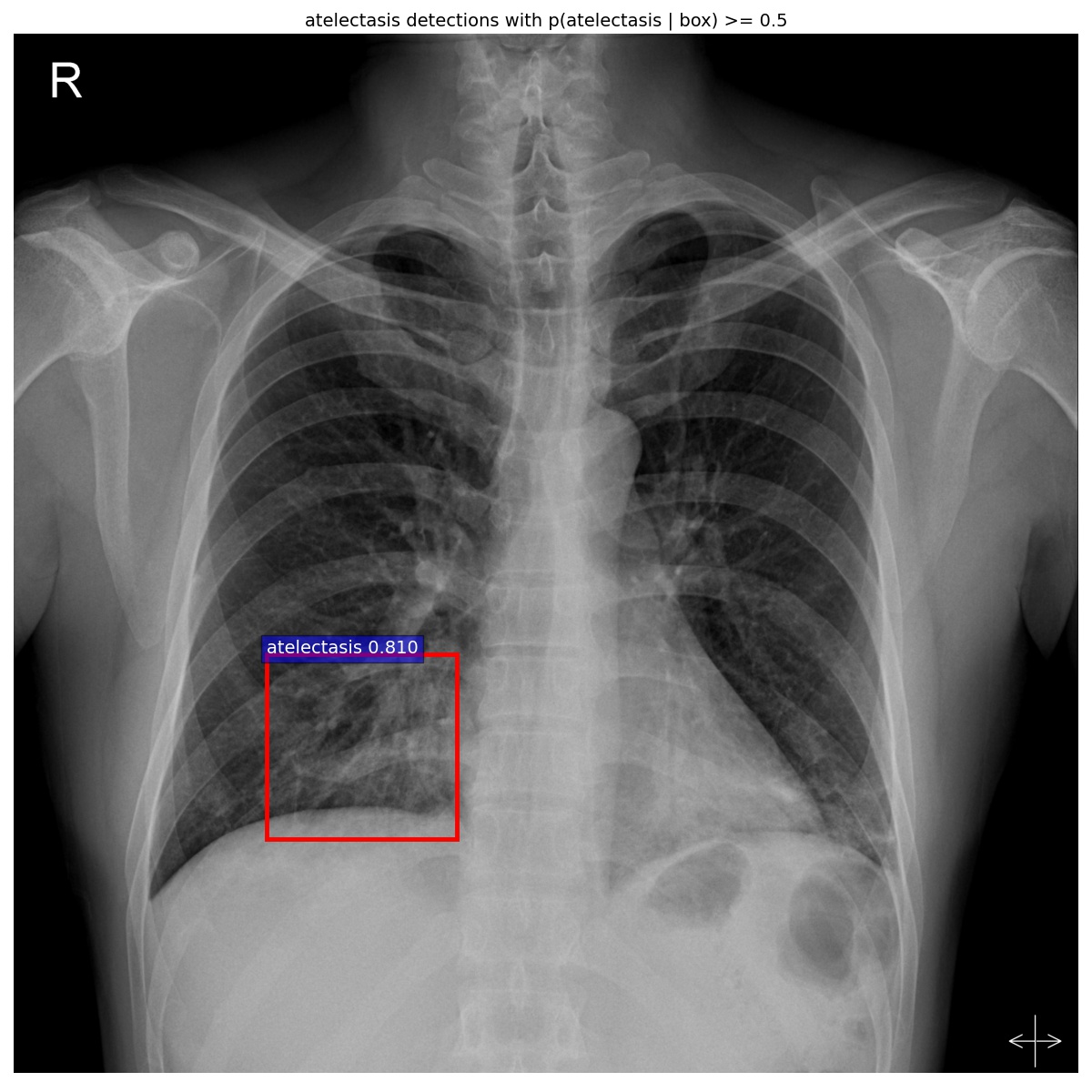


S1-3) Pulmonary nodule


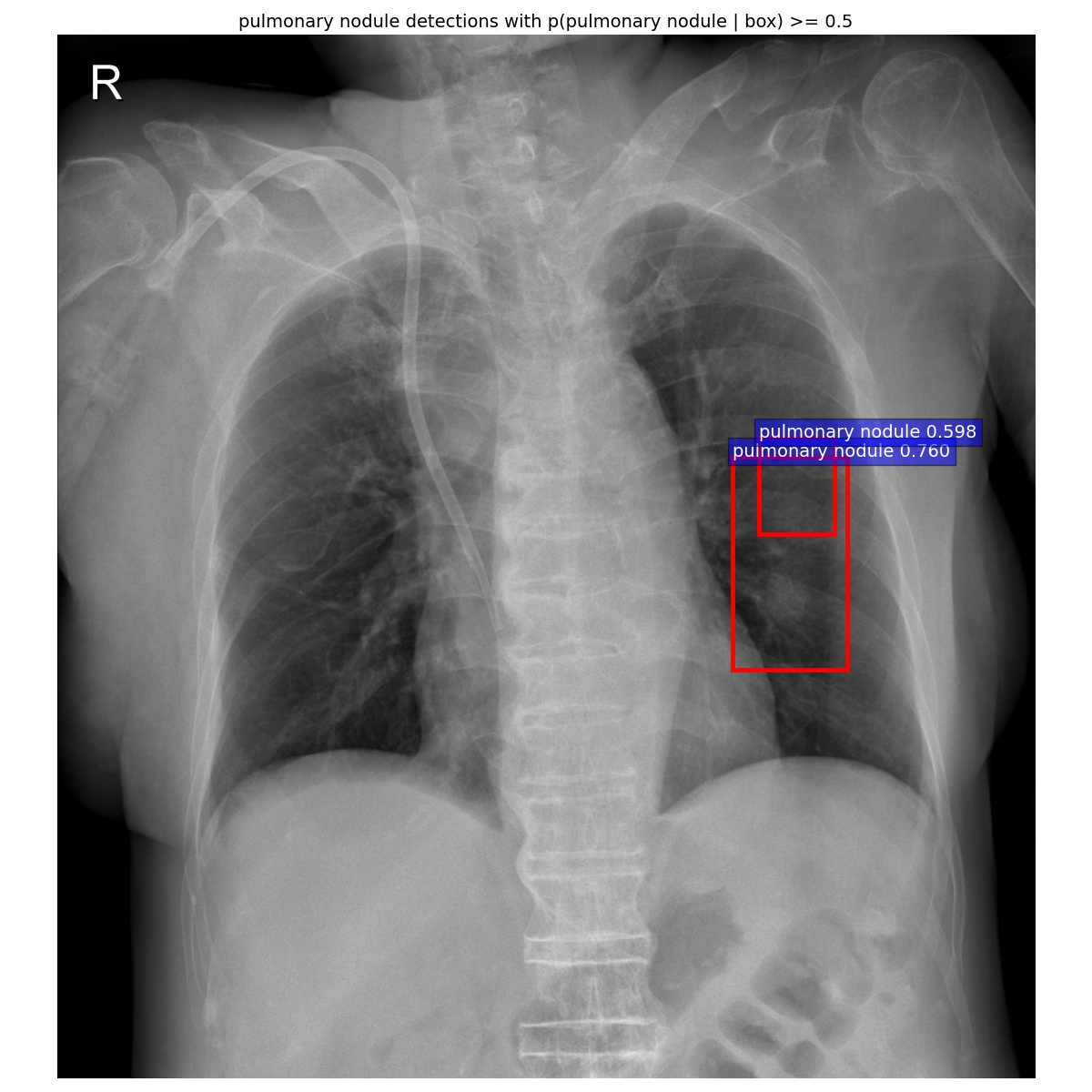


S1-4) Cardiomegaly


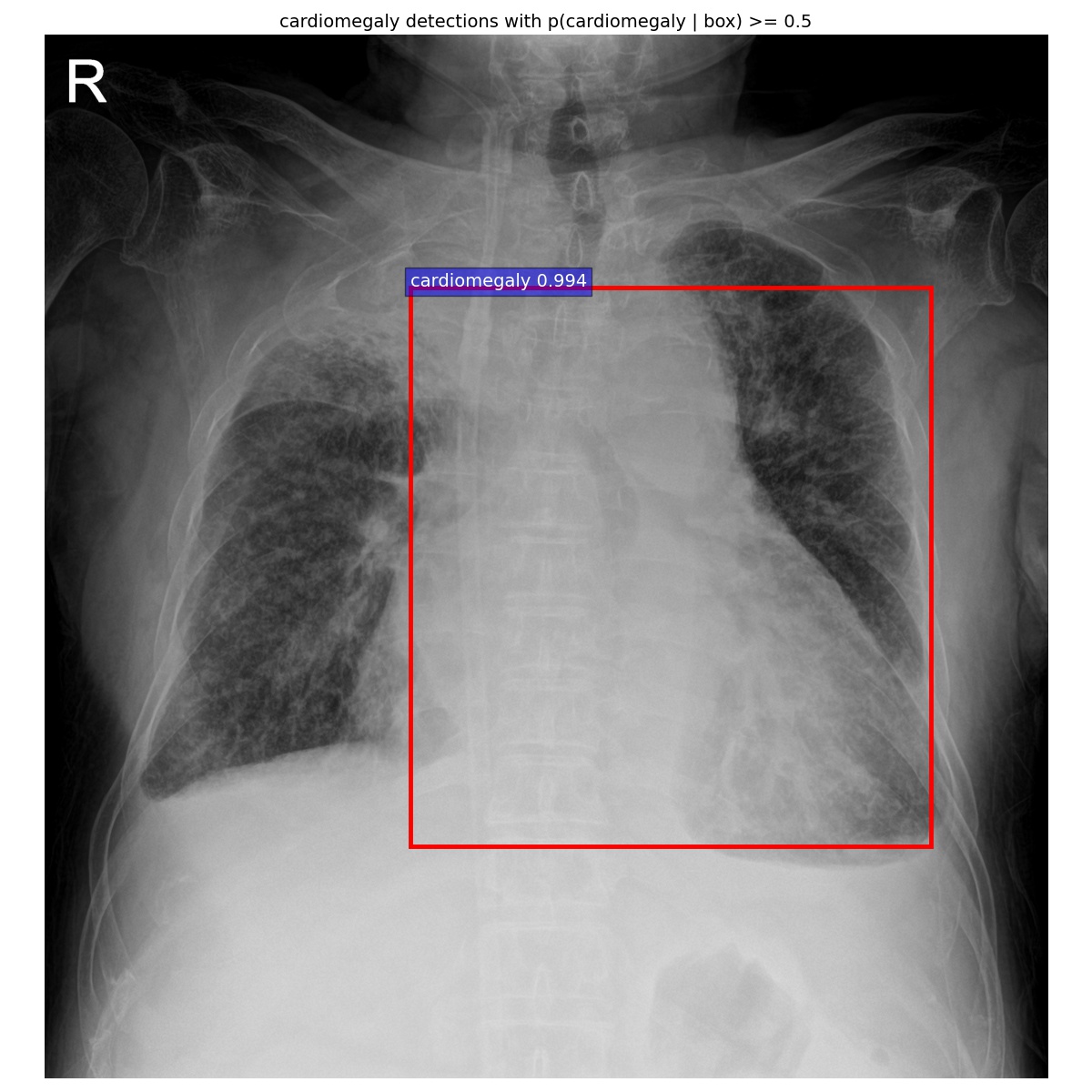


S1-5) Consolidation


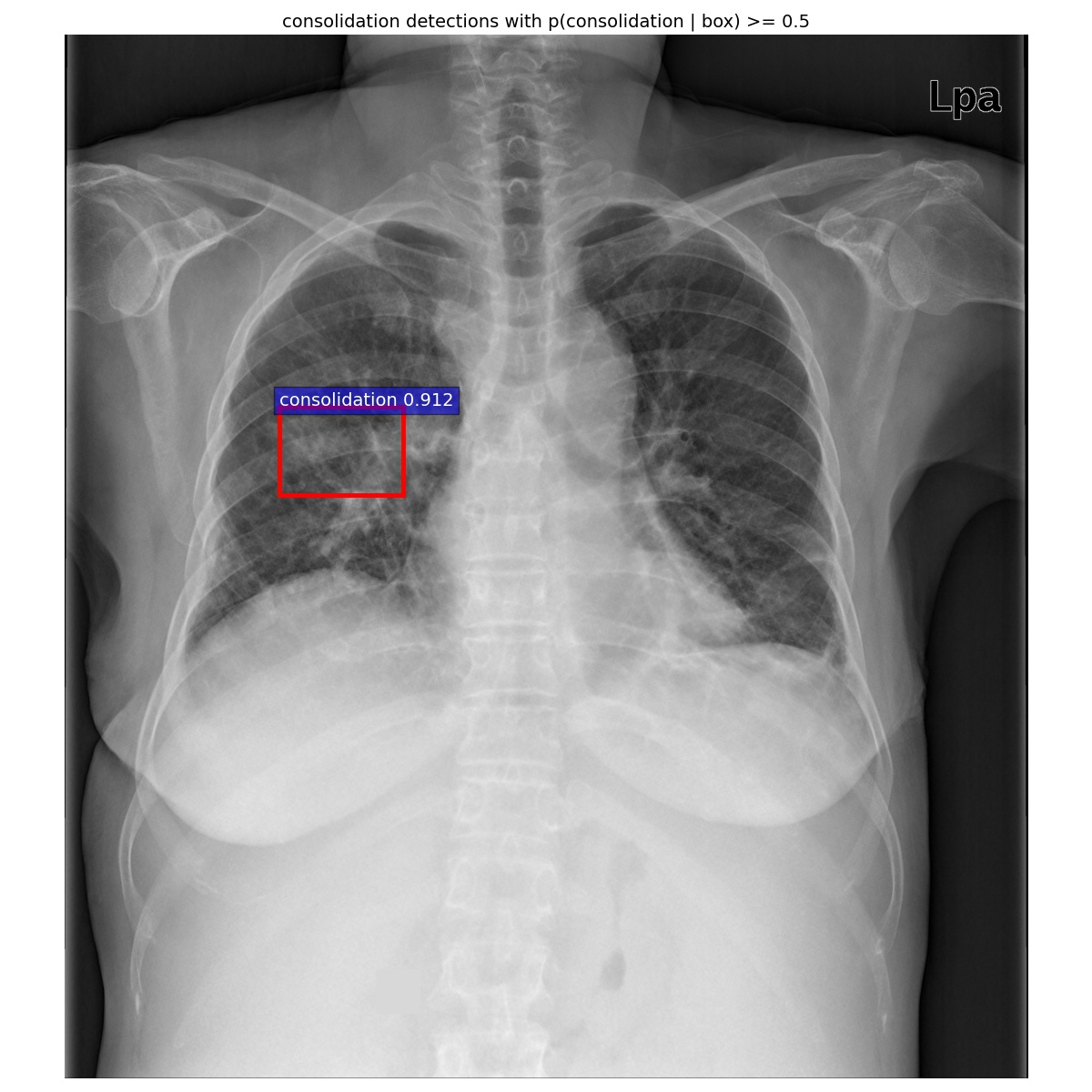


S1-6) Emphysema


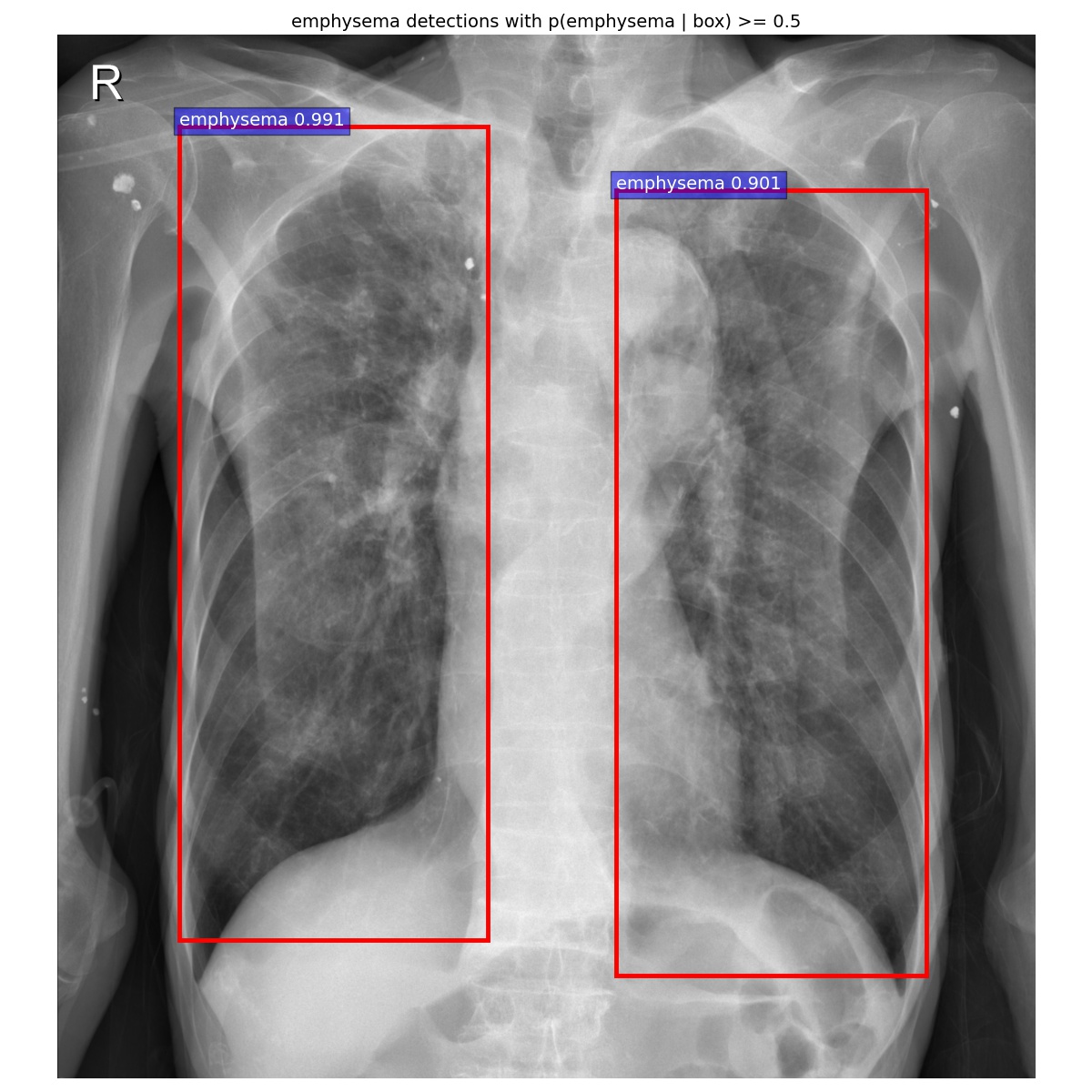


S1-7) Pneumothorax


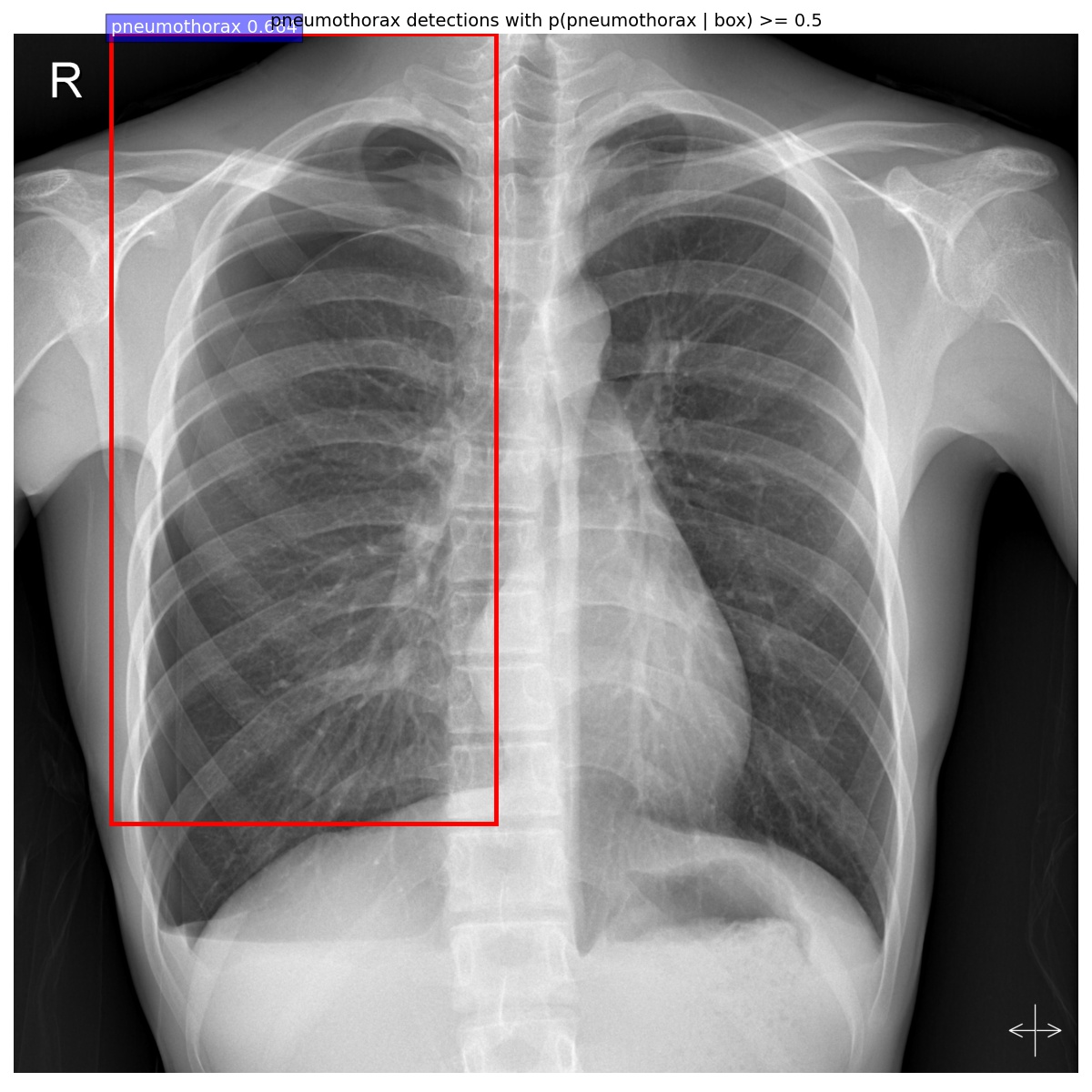


S1-8) Chemo-port


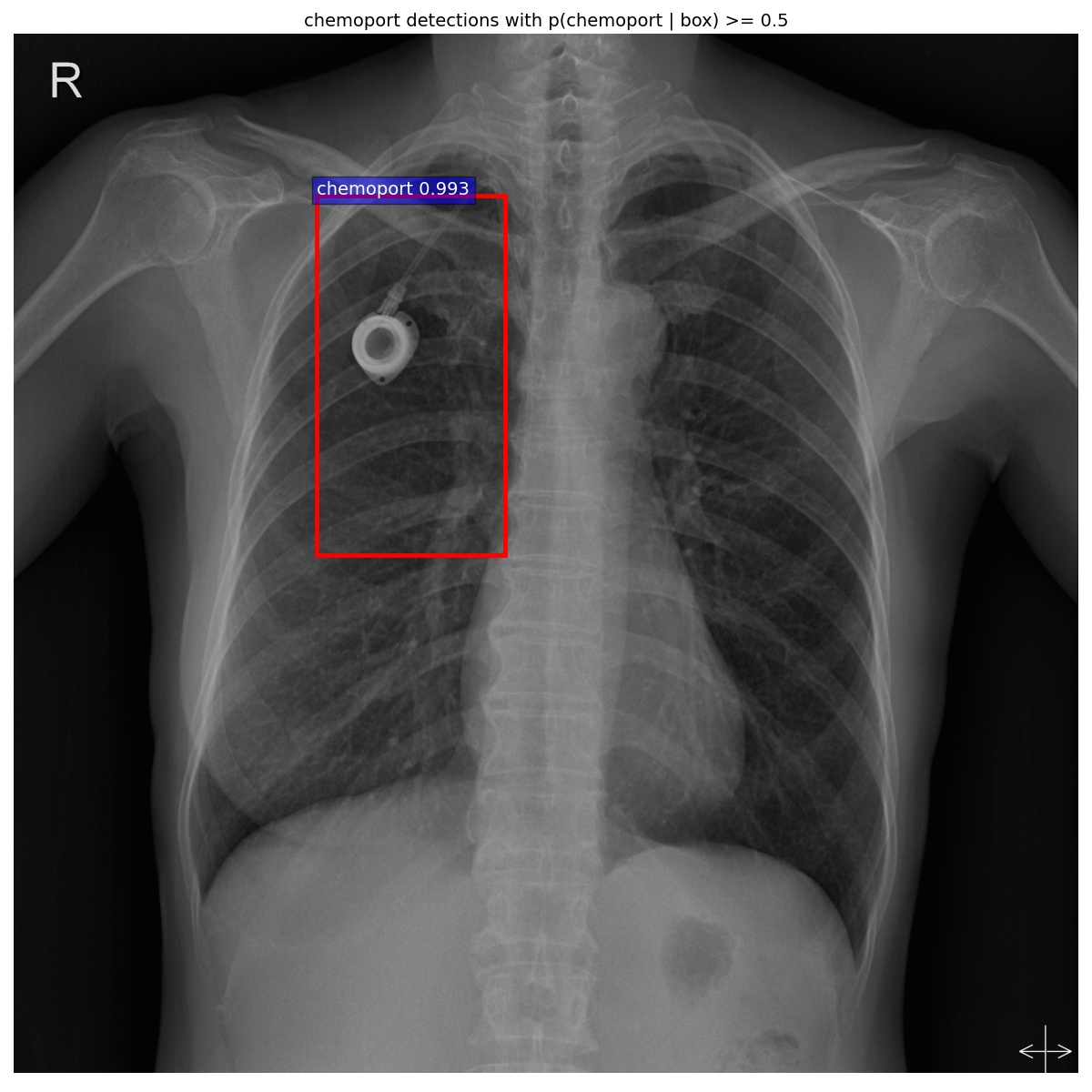


S1-9) Bronchial wall thickening


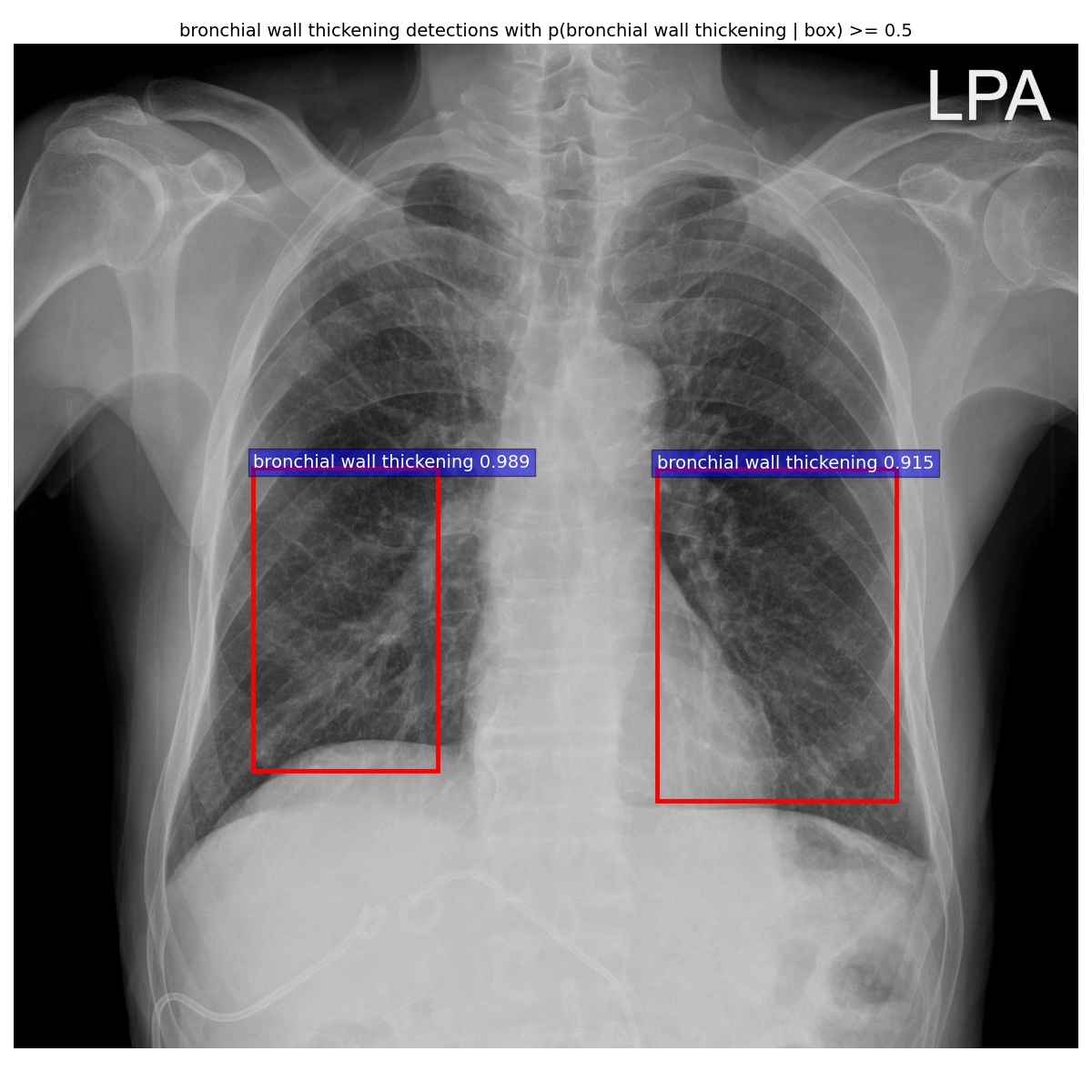


S1-10) Reticular opacities


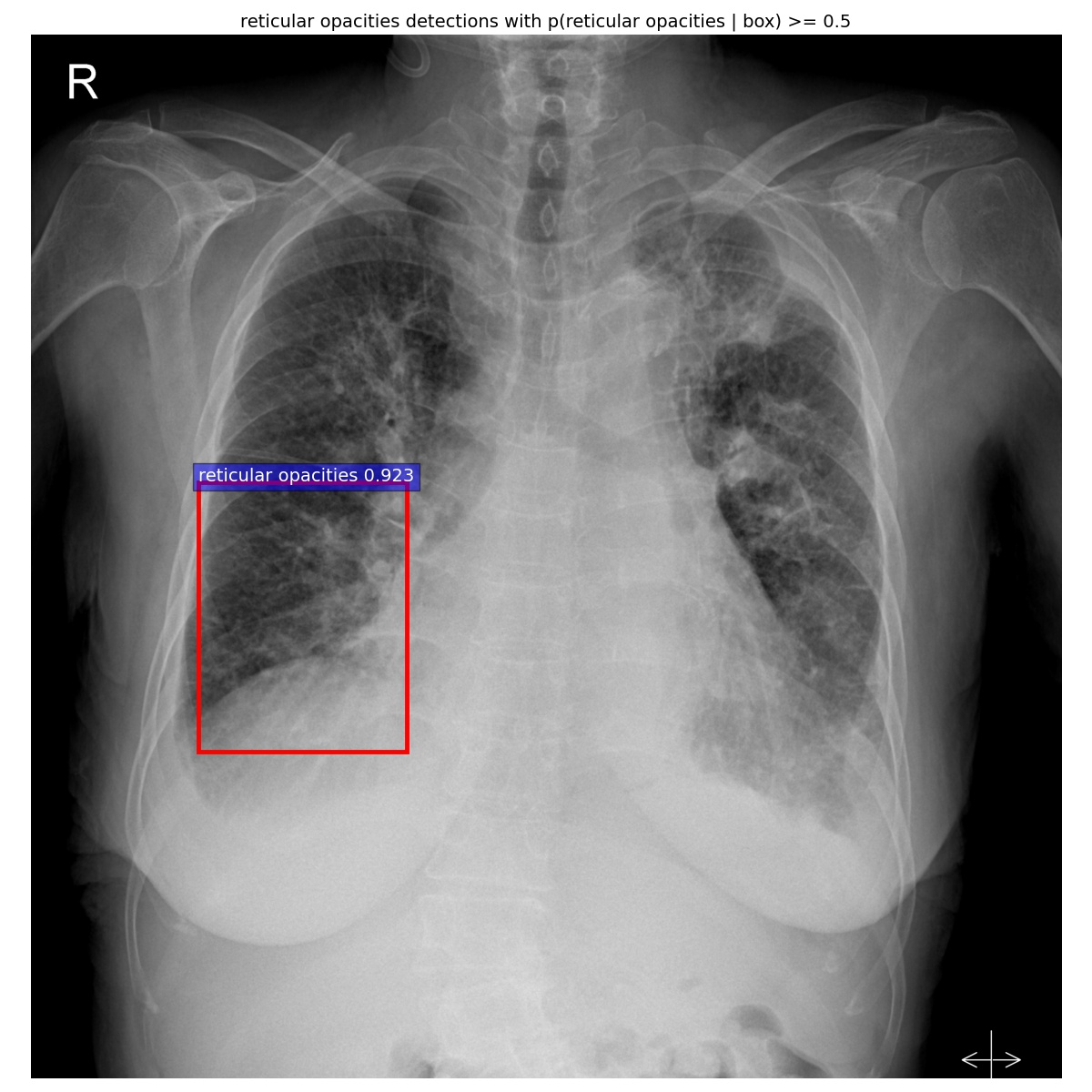


S1-11) Pleural thickening


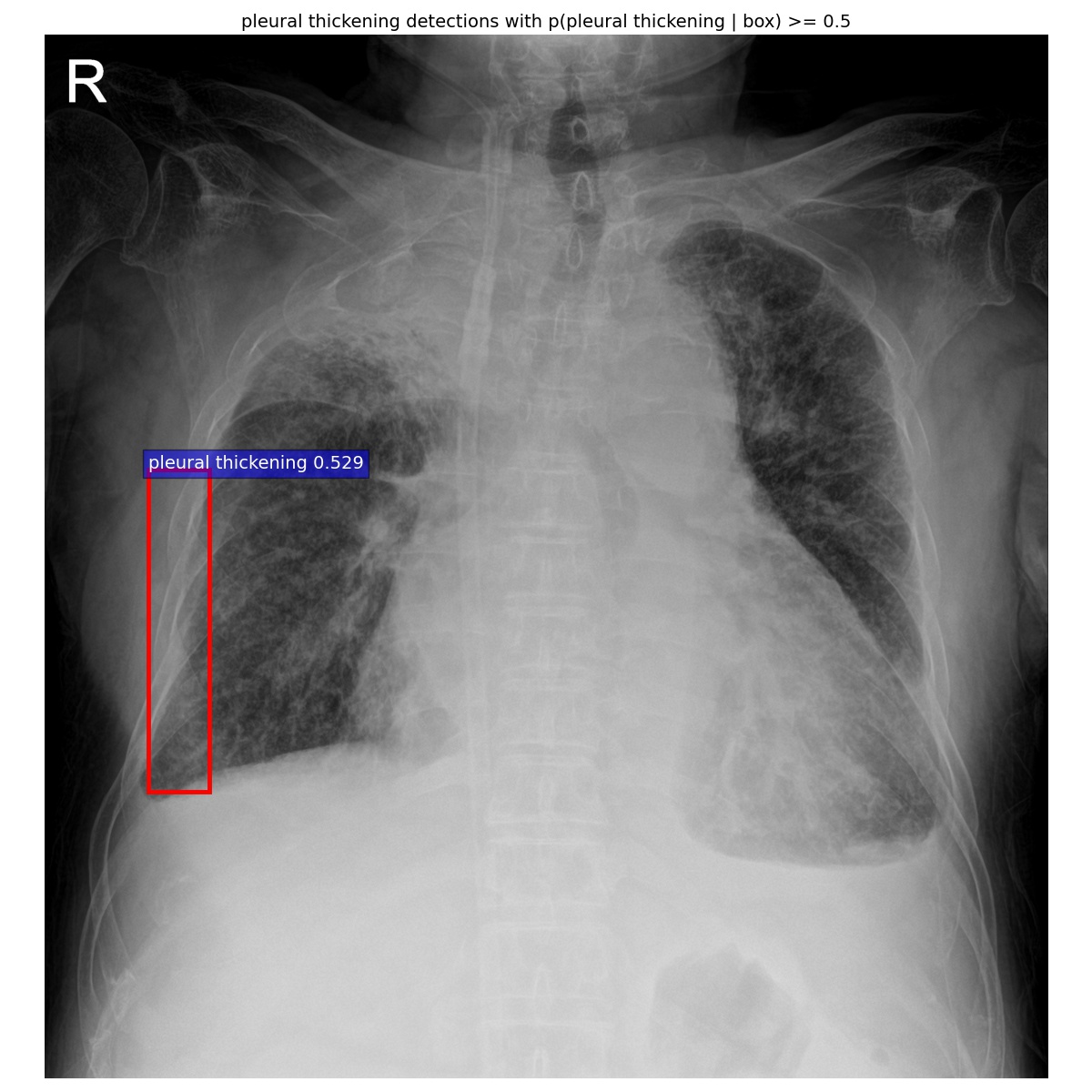


S1-12) Bronchiectasis


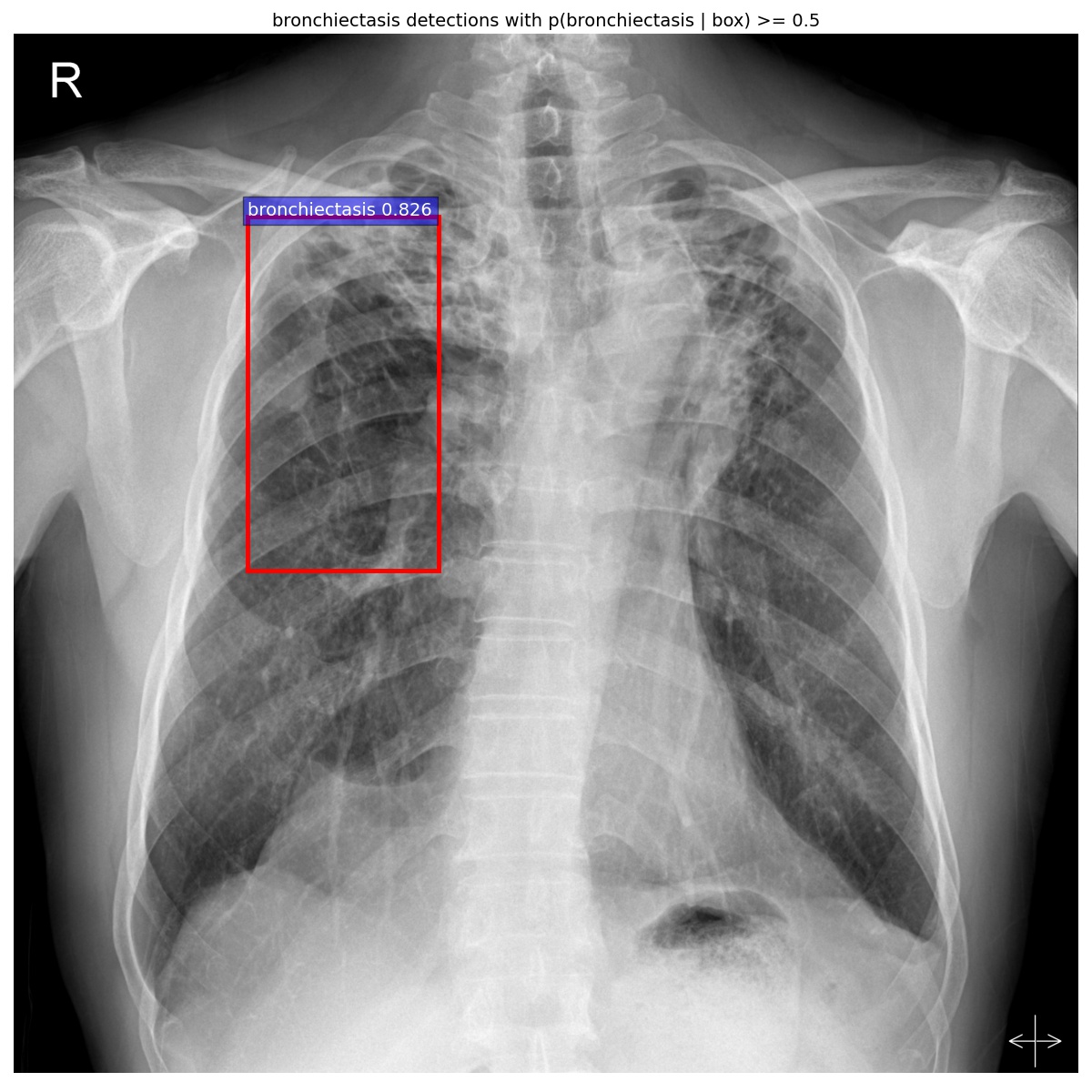

Supplement: Supplementary file 1 — Supplementary Information. [file 41598_2022_21841_MOESM1_ESM.docx]
